# Autoimmune retinopathy associated with monoclonal gammopathy of undetermined significance: a case report

**DOI:** 10.1186/s12886-020-01423-y

**Published:** 2020-04-16

**Authors:** Emily A. Eton, Gary Abrams, Naheed W. Khan, Abigail T. Fahim

**Affiliations:** 1 Kellogg Eye Center, University of Michigan, 1000 Wall Street, Ann Arbor, MI 48105 USA; 2 Kresge Eye Institute, Wayne State University, 4717 St. Antoine, Detroit, MI 48201 USA

**Keywords:** Autoimmune retinopathy, Monoclonal gammopathy of undetermined significance, Plasma cell dyscrasias

## Abstract

**Background:**

Monoclonal gammopathy of undetermined significance (MGUS) is a plasma cell dyscrasia and precursor to multiple myeloma. It has known ocular manifestations, but has not previously been shown to have an association with autoimmune retinopathy.

**Case presentation:**

A 57 year-old female presented with 1 year of progressive, bilateral, peripheral vision loss, photopsias, and nyctalopia. Her fundus examination and extensive ancillary testing were concerning for hereditary versus autoimmune retinopathy. The patient was found to have anti-retinal antibodies against carbonic anhydrase II and enolase proteins with a negative genetic retinal dystrophy panel. Malignancy work-up was negative, but the patient was diagnosed with MGUS, a premalignant condition. The patient was treated with immunosuppressive therapies, with rituximab demonstrating the most robust therapeutic response with respect to patient symptoms and ophthalmic testing.

**Conclusions:**

MGUS should be considered as a potential etiology of autoimmune retinopathy in patients without other autoimmune or malignant disease processes. Immunosuppressive therapy may be helpful in limiting disease progression, with rituximab showing efficacy in retinopathy refractory to other agents.

## Background

Monoclonal gammopathy of undetermined significance (MGUS) is a premalignant clonal plasma cell proliferation characterized by the presence of serum M-protein without associated symptoms of end-organ damage as seen with multiple myeloma (hypercalcemia, renal insufficiency, anemia, bony lesions) [1]. MGUS carries with it a risk of progression to malignancy, with 11% of MGUS patients developing multiple myeloma or another plasma-cell or lymphoid disorder [2]. Ocular manifestations of MGUS are rare but have been described in the literature and include maculopathy with serous macular detachments [3], crystalline keratopathy [4], and copper deposition in Descemet’s membrane [5]. We report a novel case of autoimmune retinopathy in a patient with MGUS.

## Case presentation

A 57 year-old female was referred with 1 year of progressive peripheral vision loss. Additional symptoms included 6 months of progressive nyctalopia and 2 months of photopsias. Her ophthalmologic history was notable for nuclear sclerotic cataracts bilaterally and a complete posterior vitreous detachment bilaterally. Medical history was significant for insulin-dependent type 2 diabetes mellitus without retinopathy and hypertension. The patient reported a family ocular history of a brother with glaucoma.

**Fig. 1:**
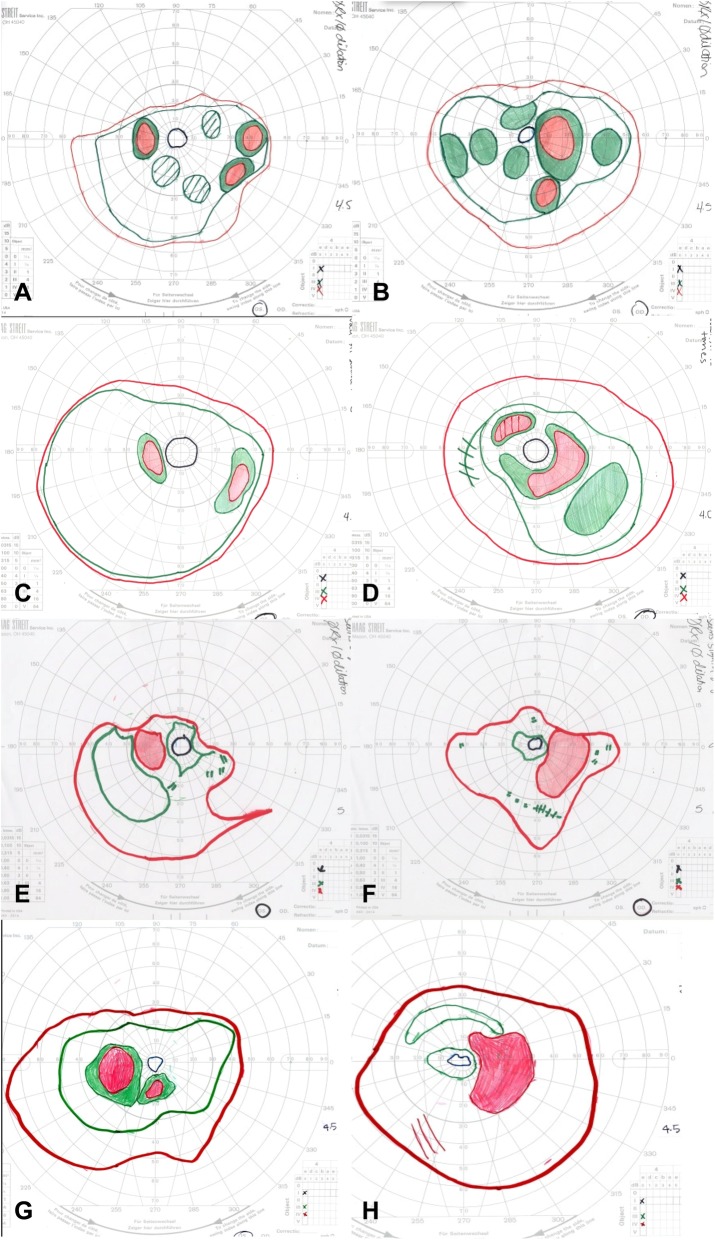
Goldmann visual fields were obtained for targets IV4e (red), III4e (green), and I4e (black). Baseline fields from the left (**a**) and right (**b**) eyes showed severe constriction of the I4e isopter and moderate constriction of III4e and IV4e isopters, with enlarged blind spots and scattered mid-peripheral scotomas bilaterally. Six months later, constriction and scotomas in the left (**c**) and right (**d**) eyes were improving on prednisone and methotrexate therapy. Five months after starting methotrexate, there was again worsening constriction of all isopters in the left (**e**) and right (**f**) eyes despite increasing methotrexate doses. The IV4e and III4e isopter constriction improved in the left (**g**) and right (**h**) eyes following initiation of rituximab infusions

Ophthalmologic exam demonstrated best corrected visual acuity of 20/25 bilaterally. Color vision testing by both Ishihara plates and Farnsworth D-15 was without deficits. Goldmann visual fields (GVF) showed moderate to severe constriction bilaterally with enlarged blind spots and scattered mid-peripheral scotomas (Fig. 1a-b). Dark adaptation threshold measured with Goldmann-Weekers dark-adaptometer was measured after 45 min in the dark and was elevated by 1.0 log unit at fixation and 2.5 log units peripherally OD, and 0.7 log units at fixation and 2.5 log units peripherally OS.

**Fig. 2:**
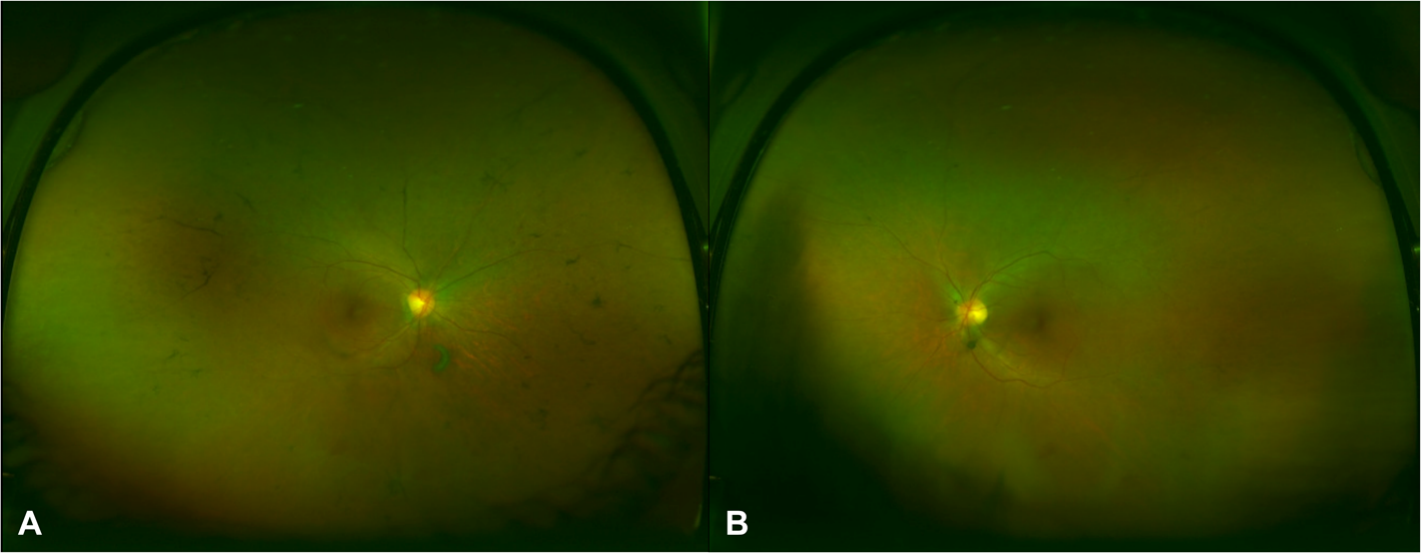
Ultra-widefield color fundus photos of the right (**a**) and left (**b**) eye demonstrated mild optic disc pallor, attenuation of retinal vessels, and mid-peripheral bone spicule pigmentation in the right eye greater than left

**Fig. 3:**
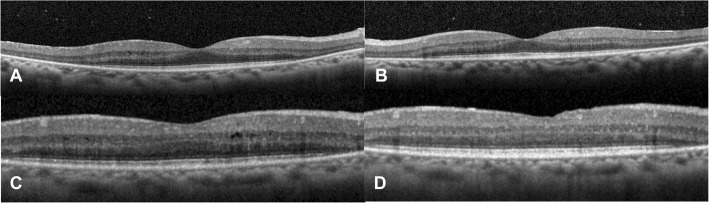
Macula OCT demonstrated preserved central island of the ellipsoid layer and outer nuclear layer in the right (**a**) and left (**b**) eyes, with otherwise severe outer retinal atrophy and trace cystic intraretinal fluid in the right eye (**c**). Six months later on prednisone and methotrexate therapy there was resolution of intraretinal fluid in the right eye (**d**)

**Fig. 4:**
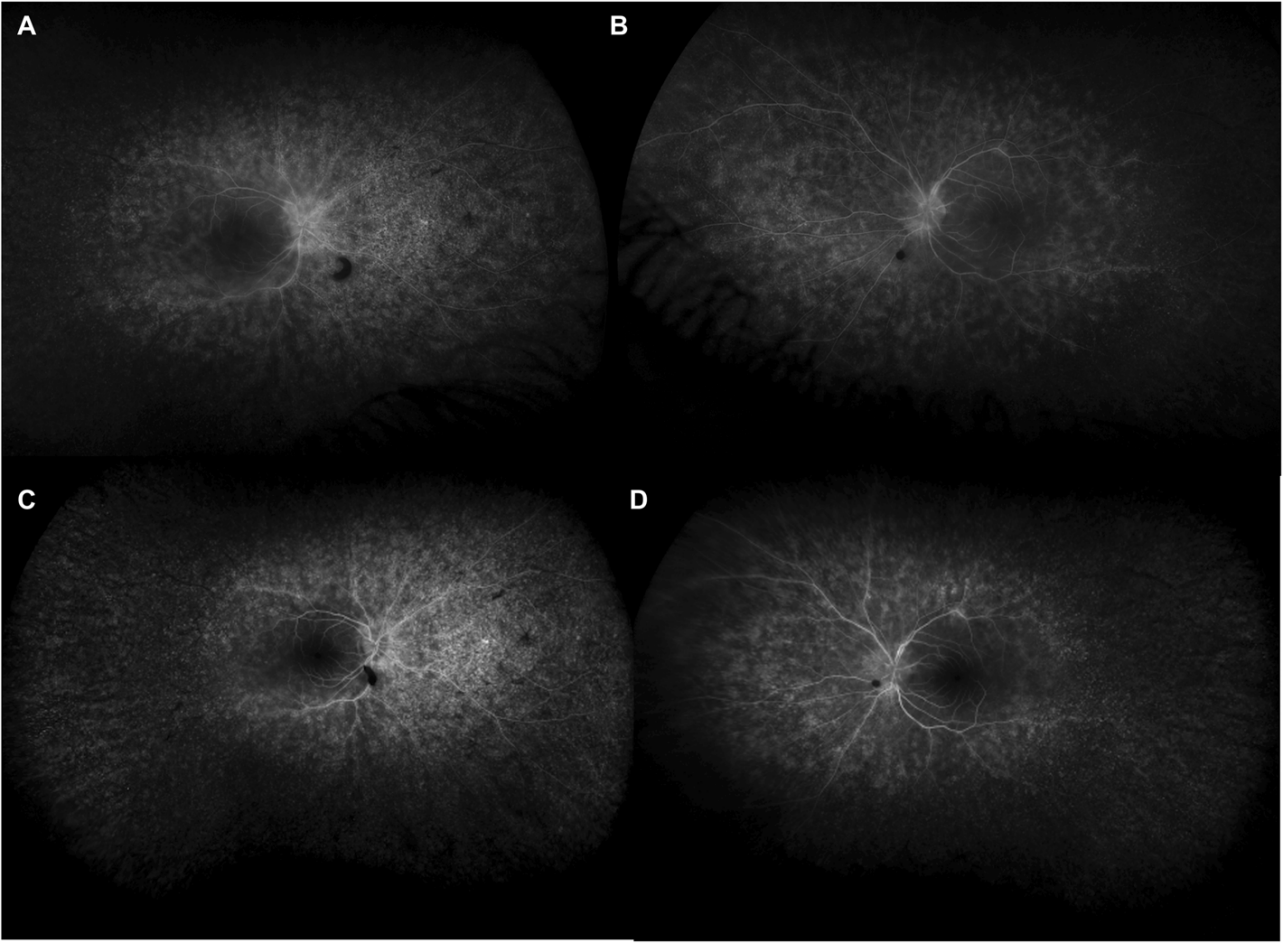
Late phase fluorescein angiography on initial presentation showed scattered areas of deep leakage with peripheral non-perfusion and disc leakage in the right (**a**) greater than left (**b**) eyes. After six months of immunosuppressive therapy there was interval decrease in leakage in the right (**c**) and left (**d**) eyes

**Fig. 5:**
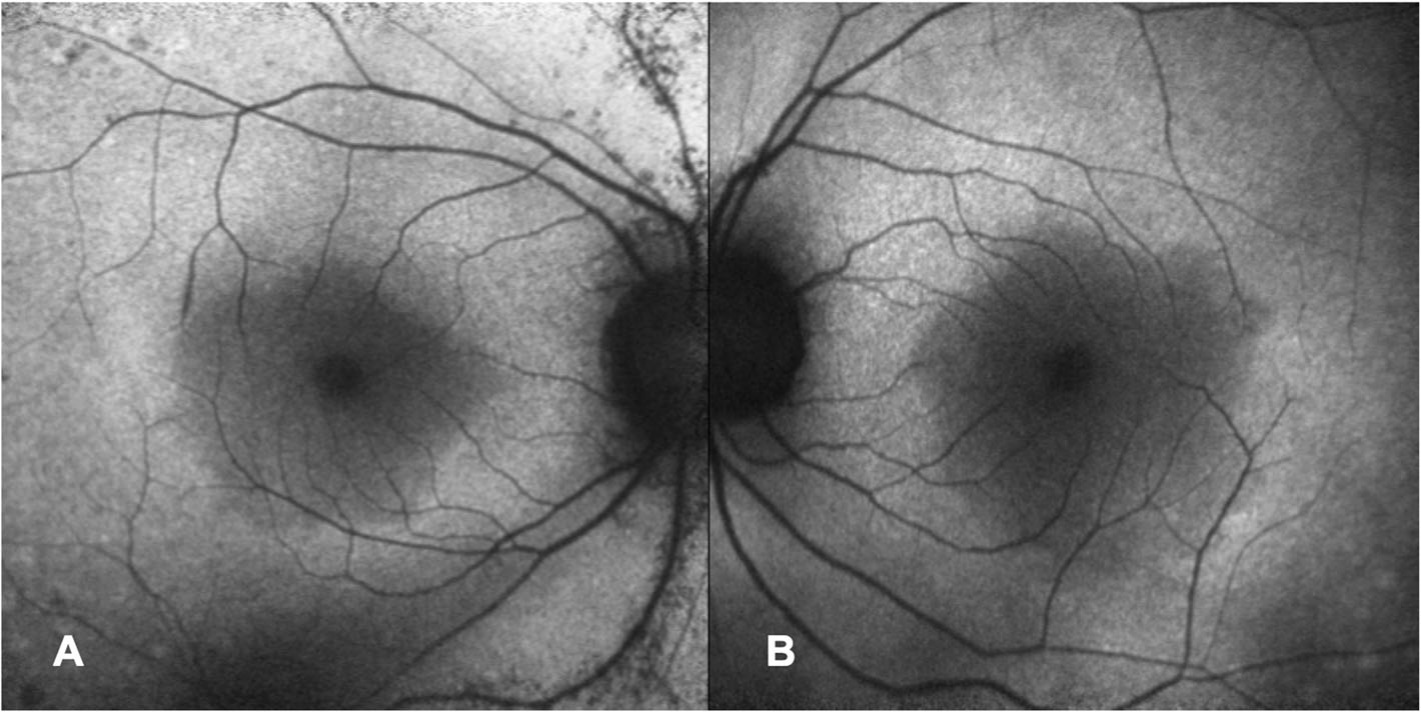
Fundus autofluorescence showed slightly increased autofluorescence circumferentially in the parafoveal region in the right (**a**) and left (**b**) eyes, with hyper- and hypoautofluorescent speckling in the right eye along the superior and inferior arcades

**Fig. 6:**
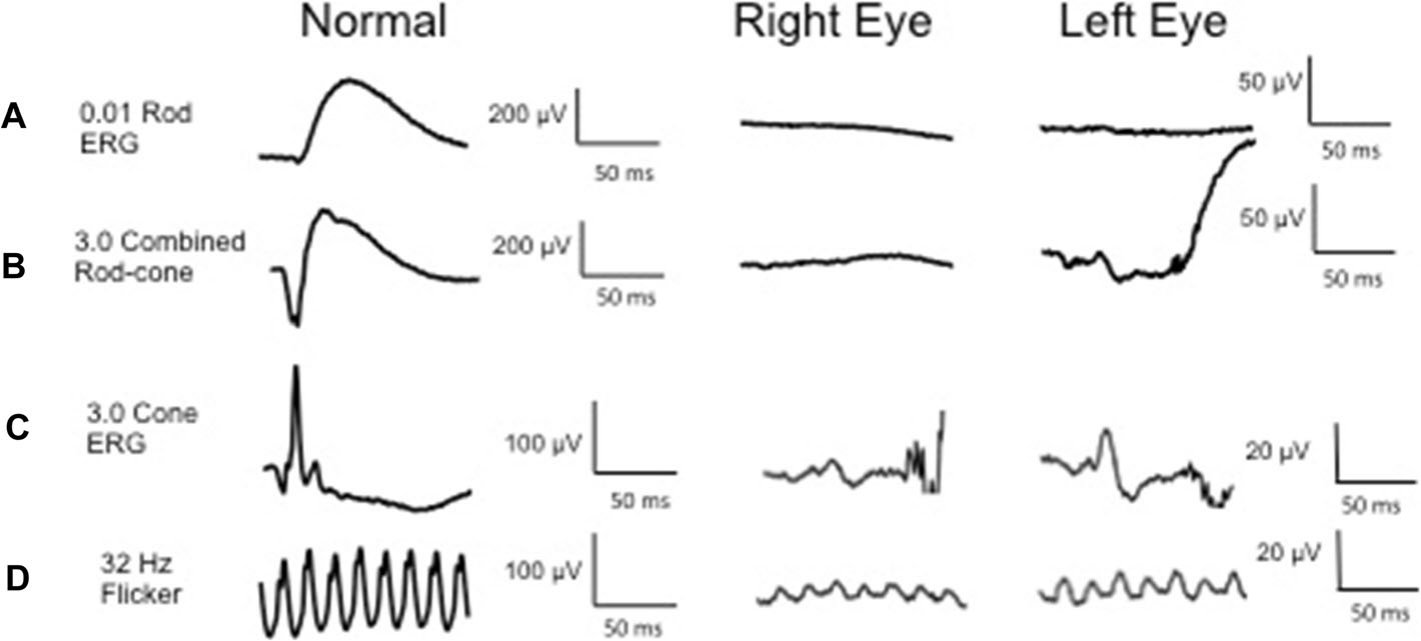
Full field electroretinography showed **a** diminished 0.1 rod isolated ERG b-wave bilaterally, consistent with severely decreased rod function; **b** scotopic 3.0 ERG with depressed combined rod-cone response in the right eye and 10% of normal a- and b-waves in the left eye; **c** residual 3.0 ERG cone response with 5% normal amplitude and delayed peak implicit time bilaterally; **d** photopic 3.0 32 Hz flicker reduced to 10% normal amplitude and delayed implicit time bilaterally

Intraocular pressure was 15 mmHg bilaterally with briskly reactive pupils without afferent pupillary defect. Slit lamp exam of the anterior segment was notable for deep and quiet anterior chambers and 1+ nuclear sclerosis bilaterally, and trace anterior vitreous cell in the right eye only. Fundus exam demonstrated Weiss rings bilaterally, mild disc pallor, and severely attenuated retinal vessels with perivascular pigment in both eyes. There was moderate bone spicule pigmentation in the mid-peripheral retina, greater in the right eye than the left eye. (Fig. 2). Macula optical coherence tomography (OCT) demonstrated severe outer retinal atrophy with central sparing of ellipsoid zone and outer nuclear layer bilaterally and trace intraretinal cystic fluid in the right eye (Fig. 3a-c). Fluorescein angiography (FA) was also obtained and in both eyes showed peripheral non-perfusion with late scattered vascular leakage and disc leakage (Fig. 4a-b). Fundus autofluorescence demonstrated diffuse hyperautofluorescence circumferentially in the parafoveal region, bilaterally (Fig. 5). Full-field electroretinography (ERG) performed with a Ganzfeld electroretinogram was recorded using the International Society for Clinical Electrophysiology of Vision protocol, and was consistent with advanced, symmetric rod-cone degeneration bilaterally (Fig. 6).

Laboratory tests, including antinuclear antibody, antineutrophil cytoplasmic antibody, c-reactive protein, complete blood count, basic metabolic panel, complement C3, complement C4, rheumatoid factor, anti-cyclic citrullinated peptide, cryoglobulin, lupus anticoagulant, angiotensin converting enzyme, lysozyme, Ebstein-Barr virus antibody, cytomegalovirus antibody, bartonella antibody, viral hepatitis panel, HIV, lyme antibody, syphilis treponemal screen, and quantiferon gold, were all negative. HLA testing was negative for HLA:A29, B51, and B27 antigens. An anti-retinal autoantibody panel ordered through the ocular immunology laboratory at the Casey Eye Institute was positive for anti-retinal antibodies against 30-kDa carbonic anhydrase II protein and 46-kD enolase protein. Serum protein electrophoresis was performed with an elevated M-spike of 0.38 g/dL (IgG lambda subtype). The patient was referred to an oncologist who diagnosed her with MGUS.

There was no family history of retinal dystrophy, and a 267 genetic retinal dystrophy panel through the Ocular Genomics Institute at Massachusetts Eye and Ear Infirmary was inconclusive. Given the relatively rapid progression in conjunction with vascular leakage on FA and the positive anti-retinal antibodies, the clinical picture was consistent with autoimmune retinopathy (AIR). Given concern for possible cancer-associated retinopathy (CAR), oncologic screening was reviewed and included a normal mammogram 9 months prior to presentation, negative fecal occult blood test 2 months prior to presentation, colonoscopy without malignant lesions 5 years prior, and normal pap smear 1 year prior. CT of the chest and abdomen and MRI of the brain and orbits were also negative for malignancy.

The patient was started on prednisone 70 mg (1 mg/kg-day) with a slow taper and mycophenolate mofetil 1500 mg twice daily was initiated within the next month. She had multiple episodes of skin peeling, which resolved after discontinuing mycophenolate. Methotrexate 15 mg weekly was initiated and the patient continued tapering prednisone down to 20 mg daily over a period of 6 months. On this regimen, she had improvement in her visual acuity, to 20/20 bilaterally, and GVF (Fig. 1c-d), resolution of macular edema on OCT (Fig. 3d), and decreased vascular leakage on FA (Fig. 4c-d). Her fundus examination remained stable. She was tapered off prednisone completely over another 4 months. One month later on methotrexate monotherapy, the patient was noted to have progressive constriction of GVF in the right eye. Methotrexate was increased to 17.5 mg weekly. Four months later, the visual acuity was 20/25 bilaterally and the patient noted a subjective decrease in peripheral vision in the right eye. Continued bilateral progression on GVF (Fig. 1e-f), return of macular edema bilaterally, and further attenuation of parafoveal ellipsoid zone in the right eye were noted. A single sub-Tenon’s methylprednisolone acetate injection (40 mg) in her right eye, the worse eye, was used to bridge the patient to rituximab infusions. After the first two rituximab infusions (1000 mg each) there was improvement in GVF constriction (Fig. 1g-h) and resolution of macular edema in both eyes. The visual acuity was 20/30 bilaterally. The patient missed her subsequent scheduled infusions due to transportation issues, and when seen 6 months after her last infusion she had a decrease in visual acuity to 20/40–1 in the right eye and 20/30–1 in the left eye, recurrence of mild cystoid macular edema bilaterally, and enlarging central scotoma in the right eye. She has recently restarted rituximab infusions. Throughout her clinical course her MGUS remained stable.

## Discussion and conclusions

MGUS is a common pre-malignant plasma cell disorder with 3.2% of patients over the age of 50 and 5.3% of patients over the age of 70 affected [6]. The disorder is characterized by a monoclonal immunoglobulin (M-protein) elevation of less than 30 g/L, bone marrow plasma cell involvement of less than 10%, and lack of sequelae attributable to multiple myeloma (hypercalcemia, renal insufficiency, anemia, or bony lesions). Patients with IgM MGUS tend to progress to Waldenström’s macroglobulinemia (WM) whereas those with IgA or IgG MGUS progress to multiple myeloma at a rate of 1% per year [7]. Our patient had a low burden of M-protein, decreasing her risk of progression to multiple myeloma, however she continues to be followed routinely by oncology with serial monoclonal protein levels.

Ocular manifestations of MGUS have been described [3–5], but to our knowledge, there are no prior reports of MGUS-associated autoimmune retinopathy. AIR is characterized by photoreceptor dysfunction and the presence of anti-retinal autoantibodies, with associated visual symptoms that may include visual field defects, decreased visual acuity, scotomas, nyctalopia, and photopsias. It can be divided into paraneoplastic and non-paraneoplastic disease processes, with the paraneoplastic subset further divided into cancer-associated retinopathy (CAR) and melanoma-associated retinopathy (MAR) [8, 9]. CAR is most prevalent in association with small-cell lung carcinoma, but has also been seen in gynecologic and breast cancers, other solid tumor malignancies, and hematologic malignancies [10, 11]. Non-paraneoplastic AIR (npAIR) patients are typically females in their 50s–60s, similar to our patient, and often have a family or personal history of autoimmune disease. Our patient reported an unspecified family member with rheumatoid arthritis. Our patient had an extensive workup, which was negative for malignancy but positive for MGUS, a non-malignant neoplastic disorder.

Though MGUS and multiple myeloma have not previously been linked to autoimmune retinopathy, cancer-associated retinopathy has been reported in the setting of WM. Liu et al. described a patient who carried a diagnosis of WM previously treated with plasma exchange, who developed scotomas, photopsias, and visual blurring [12]. Workup demonstrated depressed scotopic and photopic responses on full-field ERG with autoantibodies to 35-kDa glyceraldehyde 3-phosphate dehydrogenase as well as 43- and 44-kDa unspecified retinal proteins. Plasma exchange therapy was successful in stabilizing his cancer-associated retinopathy. Sen et al. described a WM patient who initially presented with cone dysfunction but later developed nyctalopia and showed additional rod dysfunction on ERG [13]. An autoantibody to the connecting cilium present at the inner segment/outer segment photoreceptor junction was identified on immunohistochemistry. The disease course was notably slower in progression than is often seen with CAR. Rituximab therapy did not result in clinical improvement for this patient. In both cases, the patients carried a WM diagnosis prior to developing visual symptoms, whereas our patient’s MGUS was newly identified as part of her retinopathy workup.

While WM and multiple myeloma are considered malignant disorders in contrast to MGUS, which is a non-malignant neoplastic process, all three disorders share an underlying mechanism of immune dysregulation, aberrant plasma cell proliferation, and resultant clonal antibody production. Immune dysregulation is believed to explain the associations seen between MGUS and a variety of autoimmune disorders, including ankylosing spondylitis, polymyositis, rheumatoid arthritis, polymyalgia rheumatica, pernicious anemia, and demyelinating neuropathy [14]. It stands to reason that dysregulated antibody production may underlie the development of AIR in MGUS patients, although we cannot exclude the possibility that this patient developed AIR independent of MGUS. Various retinal antibodies have been associated with AIR including autoantibodies against recoverin, inner plexiform layer, inner retinal layer, rod-transducin-α, α-enolase, and carbonic anhydrase II [15], with the latter two present in our patient.

Treatments for AIR include immunosuppressive agents aimed at decreasing circulating anti-retinal antibodies. Therapies include local or systemic corticosteroids, cyclosporine, mycophenolate mofetil, methotrexate, azathioprine, IVIG, and plasmapheresis [8, 9, 16]. There have also been reports of long-acting intravitreal fluocinolone acetonide implants being used for treatment of paraneoplastic and non-paraneoplastic AIR in patients refractory to or intolerant of systemic therapy [17]. More recently rituximab, an immunosuppressive agent targeting the CD20 protein on B lymphocytes, has been shown to be effective at stabilizing or improving clinical and laboratory markers of CAR, MAR, and npAIR in some patients [18, 19]. This was true for our patient who despite initial improvement with prednisone and methotrexate, later progressed on these therapies and has shown GVF and OCT improvement after receiving rituximab infusions. Further follow-up is needed to evaluate for a sustained response to rituximab.

This report demonstrates that MGUS should be considered in the differential diagnosis of patients presenting with autoimmune retinopathy. Importantly, ocular symptoms may be the initial presentation of MGUS, and recognition of this may allow for earlier detection and initiation of monitoring for progression to WM or multiple myeloma. Ophthalmologists should consider rituximab therapy for MGUS-associated and other forms of AIR that have been refractory to other therapies.

## Data Availability

The data from this study are available from the corresponding author on request.

## Abbreviations

AIR: Autoimmune retinopathy
CAR: Cancer-associated retinopathy
ERG: Electroretinography
FA: Fluorescein angiography
GVF: Goldmann visual field
MAR: Melanoma-associated retinopathy
MGUS: Monoclonal gammopathy of undetermined significant
npAIR: Non-paraneoplastic autoimmune retinopathy
OCT: Optical coherence tomography
WM: Waldenström’s macroglobulinemia
